# Post-Mortem Examinations in Asylums

**Published:** 1900-05-12

**Authors:** 


### Editor's Letter-Box.

[Our Correspondents are reminded that prolixity is a great bar to publication, and that brevity of style and conciseness of statement greatly facilitate early insertion.]

### POST-MORTEM EXAMINATIONS IN ASYLUMS.

Dr. H. A. Kidd, medical superintendent of the West Sussex County Asylum, referring to certain comments made by us upon the statements contained in his annual report that post-mortem examinations were made in every case of death, writes: "The comment on the same subject by the Commissioners in Lunacy in their last report upon this asylum is as follows: 'We are glad to report that post-mortem examinations were made in every case, and full records kept of the results of each examination.' The practice of making post-mortem examinations is surely the usual one in hospitals, and is of the utmost importance in an institution such as this, not merely as a means of verifying diagnosis, but in order to detect injuries which might otherwise be undiscovered, and also as a means of prosecuting pathological research in a field where so much remains to be done. The organs are not merely examined microscopically, but sections of the brain and other tissues are cut and examined by the microscope. In three instances last year results of post-mortem examinations were published in the *British Medical Journal*. I may add that the consent of the relatives is invariably procured, and I have never received a complaint of any kind upon this matter."

⁂ Clearly the extent to which the making of post-mortem examinations should be carried in any asylum must bear relation to the extent to which clinical and pathological investigations are being carried out, and if, as we gather from Dr. Kidd's letter is the case at the West Sussex County Asylum, full scientific use is being made of the opportunities offered, we quite join in the comment of the Commissioners in Lunacy. Perhaps we might go further, for there is no doubt that the point raised by Dr. Kidd as to the utility of post mortem examinations in detecting injuries which might otherwise be undiscovered is an important one.—Ed. *T. H.*

